# Farm characteristics and management routines related to cow longevity: a survey among Swedish dairy farmers

**DOI:** 10.1186/s13028-018-0390-8

**Published:** 2018-06-19

**Authors:** Karin Alvåsen, Ian Dohoo, Anki Roth, Ulf Emanuelson

**Affiliations:** 1 Department of Clinical Sciences, Swedish University of Agricultural Sciences, P.O. Box 7054, 75007 Uppsala, Sweden; 2 Department of Health Management, Atlantic Veterinary College, University of Prince Edward Island, Charlottetown, PEI C1A 4P3 Canada; 3 Växa Sverige, P.O. Box 1146, 63220 Eskilstuna, Sweden

**Keywords:** Dairy cattle, Life expectancy, Lifespan, Longevity, Management, Multiple imputation

## Abstract

**Background:**

Longevity is an important trait for increasing the profitability of dairy production. Long cow longevity is also essential to reduce the environmental impact of milk production, and to maintain positive consumer attitude. Genetic selection for increased longevity has been effective, but the phenotypic trend of longevity in Swedish dairy cows has not been improved. The objective of this study was to identify herd characteristics and management routines that are associated with the average cow longevity in a herd. To obtain this information, a questionnaire was developed and sent out to 661 Swedish dairy farmers.

**Results:**

The response rate was 35%. Seventeen of the 62 characteristics investigated had either a univariable association with the outcome (days from birth to culling) at P < 0.15, or were identified as confounders in the causal diagram and were therefore considered as candidates for the multivariable analysis. Multiple imputation was used to fill in the missing data from the questionnaires, and this increased the number of usable observations in the multivariable modeling from 156 to 228. Only a few of the investigated herd characteristics and management routines were associated with average cow longevity. The results demonstrated that using herd health advisory services shortened the average longevity, while using breeding advisory services prolonged the average longevity in the herd. Furthermore, having a greater interest in animal breeding (i.e. genetic selection) decreased the longevity, and calling the veterinarian when discovering an unhealthy cow increased the average longevity. Higher age of the farmer was also associated with longer average herd longevity.

**Conclusions:**

The herd average cow longevity was only associated with some of the farm characteristics and management routines studied. The results demonstrate that the use of advisory services and farmers’ attitudes could be targeted for increasing the herd longevity. Further, the results indicate that other e.g. qualitative factors influencing farmers’ decisions play an important role.

**Electronic supplementary material:**

The online version of this article (10.1186/s13028-018-0390-8) contains supplementary material, which is available to authorized users.

## Background

Cow longevity refers to how long the cow stays in the herd. The modern dairy cow has a short longevity, far below their biological potential. In Sweden, between 35 and 40% of the cows are culled from the herd each year, and this is done at an average age of 60.5 months [1]. Longevity is an important economic trait in dairy production [2, 3]. Increasing the productive lifetime of dairy cows would improve the efficiency of dairy production as cows must typically reach the second lactation to produce sufficient milk to break even on rearing costs [4, 5]. Herd replacement is the second highest variable cost in a dairy enterprise [6], and with increased longevity fewer replacement heifers would be required to achieve the same herd output, which would reduce replacement costs [3, 7, 8]. Surplus heifers could instead be sold or a larger proportion of the cows could be inseminated with beef semen [9]. Furthermore, with increased longevity the mean production of the herd could be higher because a large proportion of the culling decisions are based on production, and also because the proportion of mature cows, which generally produce more milk than young cows, would be increased [2, 3, 7, 10]. In addition, the carbon footprint per kilogram of product is reduced with increased lifetime productivity [11]. High culling rates and, in particular, high on-farm mortality, are indicators of poor welfare status and are not compatible with sustainable dairy production [12–14].

Longevity has been included in genetic selection in Sweden and in many other countries for many years, and a positive genetic trend has been observed (Jan-Åke Eriksson, personal communication). However, the phenotypic trend in Swedish cows has shown no improvement, and the average age at culling was still approximately 60 months in 1990 [1]. The longevity of a dairy cow is determined mainly by the culling decision made by the farmer, and such decisions are the result of intrinsic cow factors such as health, milk production, and reproductive status and extrinsic factors such as the availability of replacement heifers, the number of cow-places, the capacity of the milking system, and milk prices [15–18]. Studies have shown that pregnancy, higher milk production, younger age, and the absence of health issues such as metabolic problems, lameness, or mastitis are intrinsic cow factors that reduce the risk of culling [12, 19, 20]. On the herd level, Strandberg and Emanuelson [21] used data retrieved from the Swedish official milk recording scheme (SOMRS) and found that an expanding herd size and a high proportion of culling in early lactation were associated with both a short total lifespan and a short productive life (from first calving to culling). Management at the time around calving was suggested as a key area to improve cow longevity.

Although many intrinsic and extrinsic factors affecting longevity have been identified, very little is known about the actual herd management practices that affect these factors and thus have an impact on cow longevity. Information about such associations would be useful so that advisory activities can be more targeted. The objective of this study was therefore to identify herd management practices that are associated with average cow longevity under Swedish production conditions.

## Methods

### Study design and study population

The target population in this study was dairy farms with short or long average herd longevity (described below) and a herd size of at least 35 cows enrolled in the SOMRS. The SOMRS is a voluntary service that in 2012/2013 included 84% of the Swedish dairy cows [1]. Herds in the SOMRS are categorized into long or short longevity based on the average longevity of all cows in the herd. To avoid including herds with either temporary short or long longevity, data from three consecutive years were used. The herds were categorized into short longevity or long longevity herds based on their average longevity during 2008/2009, 2009/2010, and 2010/2011 (the SOMRS uses fiscal years running from September 1 to August 31, and this classification of year is used in the present study, with years referred to as 2009/2010, etc.). Long longevity herds had an average longevity, based on total lifespan (from birth to culling) in the fourth quartile of all SOMRS herds during 2009/2010 and 2010/2011 (1742 and 1748 days, respectively) and above the median during 2008/2009 (1579 days). Short longevity herds had an average longevity in the first quartile during 2009/2010 and 2010/2011 (1429 and 1437 days, respectively), and below the median during 2008/2009. The median was used as the threshold during 2008/2009 to increase the number of eligible herds. Out of 3698 herds with > 35 cows registered in the SOMRS, 350 and 311 herds met the criteria for short and long longevity, respectively.

### Data collection

A questionnaire was developed to acquire information on management routines that we hypothesized to be related to cow longevity in Swedish dairy herds. The questionnaire was also used in another study about on-farm cow mortality, and a more detailed description of the development of the questionnaire can therefore be found in Alvåsen et al. [14]. The questionnaire consisted of 49 questions in Swedish of which 32 were of multiple choice type, 10 were open-ended and 7 were answered on a visual analog scale. An English version of the questionnaire can be found in Additional file 1.

Questionnaires were distributed by mail in October 2012 to all farmers whose herds met the inclusion criteria. In total, 1000 questionnaires were sent, of which 661 went to the long and short longevity herds described above and 339 went to herds that were targeted for the on-farm cow mortality study. The respondents were informed that all information they provided would be treated as confidential, but they were not made aware of the study design or purpose. After 7 weeks, a reminder in the form of a postcard was sent to the non-responders. In total, 353 questionnaires were returned, of which 233 were from the 661 long and short longevity herds.

Data on average herd longevity for 2011/2012 was retrieved from the SOMRS. This average herd longevity data corresponded to the period that was covered by the questionnaire. We also retrieved data from the SOMRS on some common herd characteristics (e.g. housing and milking systems, geographic location and herd size) and average herd production parameters (e.g. milk yield and quality, reproductive performance and age at first calving) for all targeted herds. These latter data were used to compare certain parameters among respondents and non-respondents using *t* test for continuous variables and the χ^2^ test for categorical variables.

### Causal diagram

**Fig. 1:**
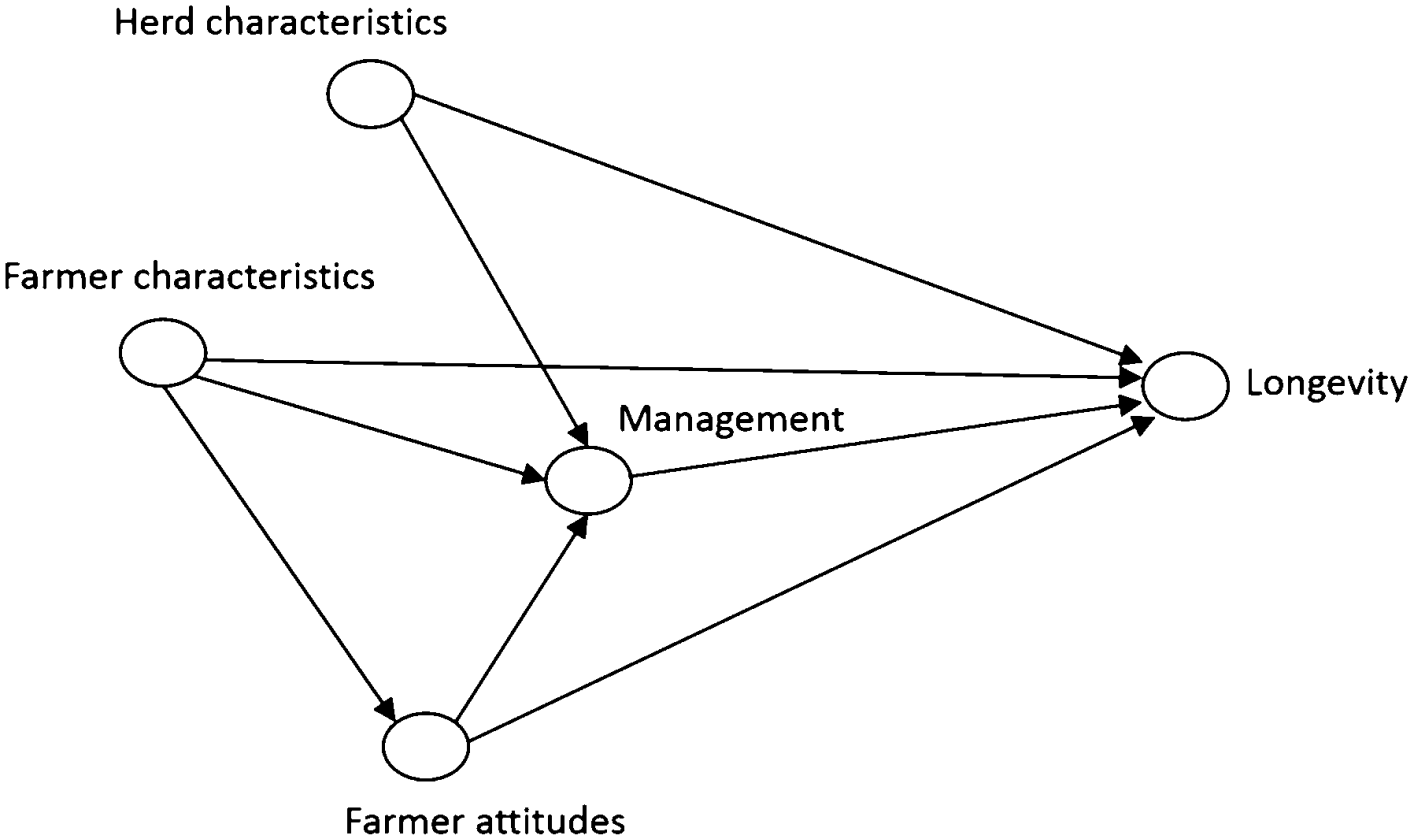
Causal diagram for analysis of the effects of farmer attitudes and management practices on longevity in dairy cows

Analyses were guided by the causal diagram shown in Fig. 1. The causal diagram was created by the authors using DAGitty [22]. Predictor variables were categorized as farmer characteristics, herd characteristics, farmer attitudes, or management practices. Variables in the latter two groups were the main exposures of interest while those in the first two groups were potential confounders. One farmer characteristic (age) and two herd characteristics (region and number of milking cows) were considered confounders in all analyses and were forced to remain in all models. Management factors were considered to be intervening variables for the evaluation of the effects of farmer attitudes and consequently were not included in these regression models.

### Outcome variable

The outcome variable of interest was the average longevity (days from birth to culling) of milking cows in the herd for the period 2011/2012. Culling was defined as the departure of cows from the herd because of sale, slaughter, salvage, or death. It was first ascertained that this variable had no missing values. Although many of the study herds had been selected based on historically long or short longevity, the variable was approximately normally distributed, although the tails of the distribution were slightly longer than expected under the assumption of normality. No transformation was employed, but the assumption of normality of residuals was checked after model building.

### Predictors

All possible predictors for which data were collected in the questionnaire were reviewed, and obvious errors were corrected. Some variables were dropped (n = 8) because there was an obvious misunderstanding of the question by some respondents (i.e., they gave completely illogical answers). Categorical variables were recoded as needed to eliminate categories with very small numbers of observations. All of the continuous variables related to farmer attitude and management factors (Table 1) were answered on a visual analogue scale where a higher number refers to “very likely” for all variables except for “Interest in animal breeding” where a high value refers to “high interest”. Visual analogue scale variables related to the diagnosis of lameness or unhealth were combined using Cronbach’s alpha into a single scale variable (an average score of standardized values). The number of valid observations for each variable was determined. A complete listing of all categorical and continuous predictors considered in the analyses (including the number of valid observations) can be found in Additional files 2, 3.

**Table 1:** Predictors selected as candidates for multivariable modeling

Variable	n	Continuous mean, SD (min, max)	Categorical		Overall
			Categories	n (%)	P-value^a^
Farmer characteristics					
Age of farmer (year)	225	48.7, 11.3 (20, 79)			< 0.001
Herd characteristics					
Herd size (no. of cows)	213	87.6, 84.6 (26, 1000)			0.192
Housing type	227		Tie stall	100 (56)	0.017
			Free stall	127 (44)	
Region	228		South Sweden	32 (14)	0.089
			East Sweden	62 (27)	
			West Sweden	74 (32)	
			Middle Sweden	20 (9)	
			North Sweden	40 (18)	
Farmer attitudes					
Expected future herd size	224		Same or fewer Expand	163 (73) 61 (27)	0.046
Expectation that the farm will still be operating in 5 year	227	7.6, 2.7 (1, 10)			0.144
Interest in animal breeding (genetic selection)	223	7.9, 2.0 (0.5, 10)			0.145
Management factors					
Composition of workforce	222		Only family members Employee(s)	94 (42) 128 (58)	0.141
Used herd health advice during the last year	182		No Yes	82 (45) 100 (55)	0.029
Used breeding advice during the last year	204		No Yes	55 (27) 149 (73)	0.123
Calving occurs in individual calving pens	227		Yes No	78 (34) 149 (66)	0.017
Time required for drying off (d)	214	7.8, 4.3 (0, 21)			0.019
Call veterinarian for lame cows	210	4.4, 3.1 (0, 10)			0.003
Move lame cows to isolation pen	202	5.3, 3.2 (0, 10)			0.029
Call veterinarian for unhealthy cows	219	7.5, 2.4 (0.1, 10)			0.011
Move unhealthy cows to isolation pen	213	6.1, 3.2 (0, 10)			0.121
Initiate treatment of unhealthy cows on my own	215	5.0, 3.4 (0, 10)			0.148

Predictors selected as candidates for multivariable modeling of associations with average herd longevity according to the categories identified in the causal diagram

^a^Univariable P-values for the confounders (age, herd size, region), but multivariable P-values for the predictors

### Unconditional associations

Associations between predictors of interest and longevity were evaluated one at a time in linear regression models in which region, herd size, and age of farmer were forced in as confounders. P-values for the associations are shown in Additional file 2. Variables with P < 0.15 were retained for use in multivariable modeling (Table 1). Following this evaluation, housing was also identified as a potentially important confounding variable and was forced into all subsequent multivariable models.

### Complete case analyses

Several multivariable linear regression models were fitted, in which region, herd size, age of farmer, and housing were forced in as confounders. Attitude variables were included in the model as potential confounders but were not forced to remain in the model if not significant. A manual backwards elimination procedure was used in which the variables with the largest P-values were sequentially removed while watching for changes in the coefficients of the retained variables. Ultimately, only management factors with P < 0.05 were retained. For the final model, graphical evaluations were used to assess the normality of residuals and to check for evidence of heteroscedasticity. The model was refitted without observations that had standardized residuals > |2.5| in order to evaluate the impact of the outliers.

The process was repeated for the evaluation of attitude variables except that management variables were excluded from the model because they were intervening variables (Fig. 1). Any attitude variables with P < 0.05 were retained in the final model.

### Multiple imputation

Multiple imputation is a flexible, simulation-based statistical technique for handling missing data [23, 24]. It has been shown that multiple imputation analyses generally produce less biased results than complete case analyses [24]. Multiple imputation of missing predictor values was carried out using the outcome variable and all predictors for which data were missing. Chained equations were used and categorical predictors were imputed using a multinomial logit function, while ordinal, dichotomous, and continuous predictors were imputed using predictive mean matching. Problems with convergence were observed for a few categorical variables, so these were converted to a set of dichotomous predictors (1 for each category). Twenty imputed data sets were generated, and diagnostic plots comparing the distribution of the observed values with the imputed values were examined for the selected predictor variables.

### Analyses of multiply imputed data

A model-building procedure (manual backwards elimination) similar to that employed in the complete case analysis was applied to the multiply imputed data. Again, separate models were built to evaluate the effects of management factors and farmers’ attitudes. A comparison of the coefficient estimates and their statistical significance was carried out for the management practice models.

All statistical analyses were performed in Stata version 14 (StataCorp LP, College Station, TX).

## Results

Of the 233 returned questionnaires, 3 herds had ceased milk production and 2 herds had sent in blank questionnaires. The number of usable questionnaires was thus 228 for a response rate of 34%. The distribution of herd characteristics was similar in the respondent and non-respondent groups (Table 2). The median of the average herd longevity of the 228 herds was 1554 days (Q1 = 1404 days, Q3 = 1752 days).

**Table 2:** Distribution of herd characteristics among respondents and non-respondents

Variable	Category	Respondents (n = 228)	Non-respondents (n = 443)	P-value^1^
Age at 1st calving (days)		866 (99.1)^2^	898 (115.3)	< 0.01
BMSCC (1000 cells/ml)		254 (85)	256 (88)	0.37
Breed^3^	SR	18.5	18.0	0.19
	SH	20.3	26.6	
	Mixed	61.2	55.4	
Calving interval (mo)		13.4 (1.1)	13.8 (1.3)	< 0.01
Herd size (cow-year)		94 (107)	88 (73)	0.79
Milk yield (kg ECM/ cow-year)		9264 (1457)	9191 (1656)	0.71
Region	North	20.2	22.4	0.60
	Middle	57.0	52.9	
	South	22.8	24.7	

Distribution of herd characteristics (percentage of observations for categorical variables and means with standard deviations for continuous variables) among respondents and non-respondents to the questionnaire

*BMSCC* bulk milk somatic cell count

^1^Significance level (*t*-test for continuous variables and χ^2^ test for categorical variables)

^2^Mean (standard deviation)

^3^SR = > 80% Swedish Red; SH = > 80% Swedish Holstein; Mixed = all other herds

### Variables and missing data

Only four predictors had complete data for all 228 herds, and 20% of the herds had complete data for all of the variables that were evaluated. The number of observations with valid data for each variable is shown in Additional file 2, 3. In general, the number of missing values for each variable was small, with a maximum of 57 missing. The variables with unconditional associations P < 0.15, as well as the confounders, are presented in Table 1 according to the groups identified in the causal diagram (Fig. 1).

### Multivariable models

Results from the final multivariable models, including the complete case and the models using imputed data, can be found in Tables 3, 4, respectively. In both models, four variables (Age, Herd size, Region, and Housing) were included as confounders. The final model with imputed data (Table 4) demonstrated that farmers that used herd health advisory services during the last year had shorter average herd longevity ( − 124 days), while farmers that used breeding advisory services had a longer average longevity (117 days). Farmers that were more likely to contact the veterinarian when detecting an unhealthy cow and farmers that did not consider themselves as interested in genetic selection had longer average longevity (23 and 21 days, respectively). The four confounders forced into the model were all non-significant except for age, where higher age of the farmer was associated with an increased herd longevity (5 days per year of age).

**Table 3:** Final complete case model of associations between management practices and average herd longevity

Variable	Coefficient	SE	P > t	95% CI
Age of farmer^a^ (year)	7.15	1.65	0.000	3.89; 10.40
Herd size^a^	− 0.03	0.22	0.887	− 0.47; 0.41
Housing^a^				
Tie stall	Reference			
Free stall	73.65	42.96	0.089	− 11.27; 158.56
Region^a,b^				
South Sweden	Reference			
East Sweden	10.02	61.68	0.871	− 111.89; 131.93
West Sweden	− 53.10	59.35	0.372	− 170.41; 64.21
Middle Sweden	− 119.62	93.33	0.202	− 304.09; 64.85
North Sweden	− 70.88	66.68	0.290	− 202.67; 60.90
Initiate treatment on my own when recognizing unhealthy cows	− 16.14	5.80	0.006	− 27.61; − 4.68
Use of breeding advisory services				
No	Reference			
Yes	108.87	44.38	0.015	21.15; 196.59
Use of preventive herd health advisory services				
No	Reference			
Yes	− 121.41	43.32	0.006	− 207.03; − 35.78
Intercept	1317.05	103.75		

Final model of associations between management practices and average herd longevity (days) from the complete case analysis based on data from 156 herds

^a^Confounders forced into the model

^b^Region: overall P-value = 0.38 (Wald test)

**Table 4:** Final model multiple imputation model of associations between management practices and average herd longevity

Variable	Coefficient	SE	P > t	95% CI
Age of farmer^a^ (year)	5.45	1.65	0.001	2.21; 8.70
Herd size^a^	0.01	0.23	0.977	− 0.44; 0.46
Housing^a^				
Tie stall	Reference			
Free stall	30.55	40.91	0.456	− 50.10; 111.19
Region^ab^				
South Sweden	Reference			
East Sweden	− 49.54	59.77	0.408	− 167.37; 68.29
West Sweden	− 104.32	57.31	0.070	− 217.30; 8.65
Middle Sweden	− 143.15	78.27	0.069	− 297.45; 11.15
North Sweden	− 140.06	64.89	0.032	− 267.98; − 12.14
Call the veterinarian when recognizing unhealthy cows	22.59	8.26	0.007	6.28; 38.91
Interest in animal breeding (genetic selection)	− 21.09	9.87	0.034	− 40.56; − 1.62
Used breeding advisory services during the last year				
No	Reference			
Yes	116.88	48.34	0.017	21.19; 212.58
Used preventive herd health advisory services during the last year				
No	Reference			
Yes	− 124.26	43.15	0.005	− 209.48; − 39.04
Intercept	1366.83	144.22		

Final model of associations between management practices and average herd longevity (days) following multiple imputation of missing data. The analysis is based on data from 228 herds

^a^Confounders forced into the model

^b^Region: overall P-value = 0.13 (Wald test)

### Model evaluation

Coefficients from the complete case and the multiple imputation models are shown in Table 5. The complete case analysis had reasonable normality and showed no evidence of heteroscedasticity. There were six observations with standardized residuals > |2.5|. Removal of these observations reduced the coefficient and significance of the variable “Use of preventive herd health advisory services”. None of the attitude variables were significant after controlling for the four confounders (data not shown).

**Table 5:** Comparison of complete case and multiple imputation models

Variable	Models with all 17 selected predictors^a^		Final models^b^	
	Complete case analysis (n = 131)	Multiple imputation (n = 228)	Complete case analysis (n = 156)	Multiple imputation (n = 228)
Confounders forced into model				
Age of farmer	4.71**	5.06***	7.15***	5.45***
Herd size	0.13	0.1	− 0.03	0.01
Housing				
Tie stall	Reference			
Free stall	95.80*	− 18.21	73.65*	30.55
Region				
South Sweden	Reference			
East Sweden	13.21	− 67.1	10.02	− 49.54
West Sweden	− 43.05	− 103.93*	− 53.1	− 104.32*
Middle Sweden	− 117.93	− 171.90**	− 119.62	− 143.15*
North Sweden	− 49.84	− 130.49*	− 70.88	− 140.06**
Predictors available for selection				
Expected future herd size				
Same or fewer	Reference			
Expand	− 48.23	− 77.56*		
Probability that the farm will still be operating in 5 year	− 7.8	− 0.94		
Interest in animal breeding (genetic selection)	− 0.6	− 16.11		− 21.09**
Composition of workforce				
Only family members	Reference			
Employee(s)	− 37.05	− 50.03		
Used preventive herd health advisory services during the last year				
No	Reference			
Yes	− 43.1	− 107.33**	− 121.41***	− 124.26***
Used breeding advisory services during the last year				
No	Reference			
Yes	86.87*	113.21**	108.87**	116.88**
Calving occurs in individual calving pens				
No	Reference			
Only individual calving pens	− 24.02	− 60.42		
Average time for dry-off (d)	− 1.11	1.69		
Call veterinarian when recognizing a lame cow	8.62	6.04		
Move to isolation pen when recognizing a lame cow	− 7.14	− 9.89		
Call veterinarian when recognizing an unhealthy cow	15.08	16.70*		22.59***
Move to isolation pen when recognizing an unhealthy cow	9.39	5.12		
Initiate treatment on my own when recognizing unhealthy cows	− 8.79	− 4.69	− 16.14***	
Intercept	1295.35***	1483.90***	1317.05***	1366.83***

Comparison of full and final models from both the complete case and the multiple imputation models of associations between management practices and average herd longevity (*P < 0.1, **P < 0.05, ***P < 0.001)

All missing values were successfully imputed, and comparison of the distribution of imputed and original values showed good imputation. We used the coefficient of determination (R^2^) statistic to compare the predictive abilities of the complete case and the multiple imputation procedures. The two procedures produced different final models, but regardless of whether the comparison was based on applying each procedure to the complete observations (n = 156) or to a larger subset where the variables in the two models had complete information (n = 182), the model derived from the imputation procedure had better predictive ability (R^2^ = 26.2% vs. 24.2% if n = 156, ${\text{R}}_{{ ( {\text{average}}\,{\text{d}}\,{\text{over}}\, 2 0\,{\text{imputations)}}}}^{2} = 21.4\% \,{\text{vs}}\,18.1\% \,{\text{if}}\,{\text{n = 182}}$).

## Discussion

This study aimed to identify herd management practices and characteristics associated with average cow longevity in the herd. We found surprisingly few predictors that had a significant impact on cow longevity. Herds that are using herd health advisory services were associated with shorter cow longevity, but this does not necessarily imply that dairy farms should not use herd health advisory services. Instead, these services might be used to a higher degree by farms with herd health problems and thus possibly also shorter longevity. In any cross-sectional study (such as this) it is difficult to sort out the time sequence between predictors and the outcome of interest. In some situations, it is necessary to use strategies that involve higher culling rates, e.g., when combating aggressive pathogens in a herd [25, 26]. This approach can result in improved herd health in the future, but will result in a higher culling rate and hence reduce the average longevity of the herd in the short term. Also, preventive herd health services offered by veterinarians or other advisors in Sweden are not fully recognized among the dairy farmers. In a qualitative study about preventive herd health management, Swedish dairy farmers had difficulties in defining the concept of preventive herd health and in telling what roles the veterinarian could fill in this regard [27]. One key barrier for using veterinarians more proactively in herd health management that has been mentioned by farmers is that veterinarians are associated only with their curative role in acute illness [28, 29]. Thus there is a great need for veterinarians to communicate and improve the herd health services being offered—both in Sweden and in some other countries—in order to change the current stereotype of being seen as “fire fighters” who are only treating unhealthy cows [29, 30]. These difficulties for farmers in defining the concept of preventive herd health management, along with the limited experience of this type of advisory service, could have introduced observational bias in our data.

Using breeding advisory services was, however, beneficial for the average cow longevity even though being interested in animal breeding reduced the average longevity. This suggests that farmers with low interest in animal breeding might be the ones using breeding advisory services, which might result in having a more structured breeding plan and thus more robust cows. Farmers interested in animal breeding might also have a more “active” culling strategy and hence end up with a shorter average lifespan in the herd. In face-to-face interviews with Swedish dairy farmers, Bergeå et al. [31] discovered a management-related phenomenon where farmers interested in genetic selection and breeding improvements are more likely to accept that heifers “push out” older cows. This was mainly a consequence of farmers breeding too many heifers combined with a greater interest in genetic improvements. The difference in genetic potential has been shown in Swedish dairy herds; for example, herds with the lowest average estimated breeding value in milk were four times more likely to have a long average productive life and total life span than average herds [21]. One way to extend the average cow longevity is to bring fewer heifers into the herd and therefore to cull fewer cows [8, 15]. One management option would be the use of sexed semen with genetically superior cows and inseminating a proportion of the remaining cows with beef semen—which will also improve the farm economy [9]. It is likely that farms taking breeding advice receive support in these kinds of breeding strategies as genetic improvement is important but does not warrant short cow longevity [32].

Moreover, the results of the present study indicate that farmers that are more likely to contact the veterinarian as a first step when recognizing an unhealthy cow were associated with longer average cow longevity. Prompt and adequate treatment is in most situations crucial to solving health issues and thus reducing the need for premature culling. Also, achieving greater longevity through improved cow health will improve cow welfare [32].

The only confounder in the model that was statistically significant was the age of the farmer, and older farmers generally had a longer average longevity in their herds. The age of the farmer and the farmer’s experience were highly correlated, and having greater experience in running a dairy enterprise appears to improve longevity. An older and more experienced farmer might be better at keeping the animals healthy, which will reduce the number of forced culls. Another suggestion is that an older farmer might approach dairying as a lifestyle rather than a business, and therefore have a different view on ownership of their cows that transcends economic decision-making and rationale culling. Younger farmers could be more economically driven and therefore have a higher interest in genetic and herd heath improvement which could result in a shorter average cow longevity.

In this study we used two different models: one complete case analysis and one analysis of the data material that had undergone multiple imputation. The complete case analysis had somewhat different variables in the final model. The use of breeding and herd health advice services was similar to the multiple imputation model, and the directions of the effects were the same. However, instead of calling the veterinarian when discovering a sick cow, this data set showed a negative association between the average longevity of the herd and farmers that most likely “Initiated treatment on one’s own” when discovering an unhealthy cow. As multiple imputation analyses generally produce less biased results than complete case analyses [24], more weight is put on the results from the multiple imputation model. It is generally recommended to use a large number of predictors in the imputation process, and in this case we used all available data from the questionnaires. When comparing R^2^ on the same number of observations for the two models, the gain in explanation was rather small, but an important benefit with the imputation is that it increased the number of usable observations from 156 to 228. Being able to use the data from these observations most likely contributed to less biased results in this study.

The questionnaires were sent to herds in the lowest and highest quartiles of average longevity. Our initial intention was to use these classifications for the analyses, but instead we retrieved additional data from the SOMRS on average longevity for the responding herds. In this way we were able to analyze the actual average herd longevity for the same period in which the farmers filled out the questionnaire. Even though the selection criteria were based on three consecutive years, there were some herds that had changed group, and basing the analysis on the currently used farm characteristics was therefore considered to give more valid results regarding management routines and average longevity of the herd.

Because not all of the farmers responded to the questionnaire, selection bias might have been present. However, when comparing some of the herd characteristics, only two characteristics differed between respondents and non-respondents. Respondents had herds with significantly lower age at first calving and shorter calving intervals, which could reflect the level of management. Most herd characteristics showed no differences between respondents and non-respondents, and we therefore believe that the study population was reasonably similar to the target population and thus had an acceptable level of external validity.

We did not find many management-related associations, which was contrary to our expectation. Either we did not have the correct questions in our questionnaire, or a questionnaire is not the best way to capture the type of factors that we were interested in, or other unknown factors are more important, or there simply are not many management-related associations. Fetrow et al. [33] point out that a short lifespan of a dairy cow is primarily the result of an economic decision on the part of the dairy farmer and making a decision to replace one cow with another could sometimes be a strategic economic option. Different herd conditions will most likely play a significant role in culling decisions, and characteristics such as the availability of heifers, cultural influences, farmer’s attitude, and prices on the milk and beef market are some of the possible factors that have an impact on the decisions taken by the farmer [34]. Furthermore, using averages on herd level for a trait like longevity could cause bias because the same averages may come from herds with vastly different age patterns.

Longevity has been ranked as the most important trait by both organic and conventional Swedish dairy producers [35]. The longevity-related decisions taken by dairy farmers are however complex to understand, and longevity is not usually visible in farm-management data. Even though the average herd longevity is highly dependent on the decisions taken by the individual farmer, farmers themselves have expressed a low level of consciousness of the power they have to influence the longevity in their herd [31]. These kinds of qualitative factors are difficult to capture in a questionnaire of the type used in this study, but they might have a significant influence on the longevity of dairy herds.

## Conclusions

The present questionnaire study identified only a few predictors that were associated with average longevity in Swedish dairy herds. This indicates that there might be other important factors that influence herd longevity and that more effort is needed to identify which herd-specific actions need to be taken by Swedish farmers to increase the productive life of dairy cows.

## Additional files

**Additional file 1.** An English version of the questionnaire that was sent to Swedish dairy farmers.

**Additional file 2.** Continuous variables used in the analysis of factors related to average herd longevity in 228 dairy herds in Sweden. Descriptive statistics and P-values of associations with herd average longevity are presented.

**Additional file 3.** Categorical variables used in the analysis of factors related to average herd longevity in 228 dairy herds in Sweden. Descriptive statistics and P-values of associations with average herd longevity are presented.

## References

[1] Växa Sverige. Cattle statistics. 2017. http://www.vxa.se/fakta/styrning-och-rutiner/mer-om-mjolk. Accessed 28 Aug 2017.

[2] Allaire FR, Gibson JP (1992). Genetic value of herd life adjusted for milk production. J Dairy Sci.

[3] Pritchard T, Coffey M, Mrode R, Wall E (2013). Understanding the genetics of survival in dairy cows. J Dairy Sci.

[4] Archer SC, Mc Coy F, Wapenaar W, Green MJ (2013). Association between somatic cell count early in first lactation and the longevity of Irish dairy cows. J Dairy Sci..

[5] Boulton AC, Rushton J, Wathes DC (2017). An empirical analysis of the cost of rearing dairy heifers from birth to first calving and the time taken to repay these costs. Animal..

[6] Chamberlain T (2012). Understanding the economics of dairy farming Part 1: income, costs and profit. Livestock..

[7] Brickell JS, Wathes DC (2011). A descriptive study of the survival of Holstein-Friesian heifers through to third calving on English dairy farms. J Dairy Sci..

[8] Kelleher MM, Amer PR, Shalloo L, Evans RD, Byrne TJ, Buckley F, Berry DP (2015). Development of an index to rank dairy females on expected lifetime profit. J Dairy Sci..

[9] Ettema J, Thomasen J, Hjorto L, Kargo M, Ostergaard S, Sorensen A (2017). Economic opportunities for using sexed semen and semen of beef bulls in dairy herds. J Dairy Sci..

[10] VanRaden PM, Wiggans GR (1995). Productive life evaluations: calculation, accuracy, and economic value. J Dairy Sci..

[11] Knapp JR, Laur GL, Vadas PA, Weiss WP, Tricarico JM (2014). Invited review: enteric methane in dairy cattle production: quantifying the opportunities and impact of reducing emissions. J Dairy Sci..

[12] Weigel KA, Palmer RW, Caraviello DZ (2003). Investigation of factors affecting voluntary and involuntary culling in expanding dairy herds in Wisconsin using survival analysis. J Dairy Sci..

[13] de Vries M, Bookers EAM, Dijkstra T, van Schaik G, de Boer IJM (2011). Invited review: associations between variables of routine herd data and dairy cattle welfare indicators. J Dairy Sci.

[14] Alvåsen K, Roth A, Jansson Mörk M, Hallén Sandgren C, Thomsen PT, Emanuelson U (2014). Farm characteristics related to on-farm cow mortality in dairy herds: a questionnaire study. Animal..

[15] De Vries A. Cow longevity economics: The cost benefit of keeping the cow in the herd. In: Proc. of the cow longevity conference. 2013. http://www.milkproduction.com/Library/Editorial-articles/Cow-Longevity-Conference-2013—proceedings. Accessed 15 June 2016.

[16] Rajala-Schultz PJ, Gröhn YT (1999). Culling of dairy cows. Part II. Effects of diseases and reproductive performance on culling in Finnish Ayrshire cows. Prev Vet Med..

[17] Rajala-Schultz PJ, Gröhn YT (1999). Culling of dairy cows. Part III. Effects of diseases, pregnancy status and milk yield on culling in Finnish Ayrshire cows. Prev Vet Med..

[18] Schneider MDP, Strandberg E, Emanuelson U, Grandinson K, Roth A (2007). The effect of veterinary-treated clinical mastitis and pregnancy status on culling in Swedish dairy cows. Prev Vet Med..

[19] Pinedo PJ, De Vries A, Webb DW (2010). Dynamics of culling risk with disposal codes reported by dairy herd improvement dairy herds. J Dairy Sci..

[20] Rajala-Schultz PJ, Gröhn YT (1999). Culling of dairy cows. Part I. Effects of diseases on culling in Finnish Ayrshire cows. Prev Vet Med..

[21] Strandberg E, Emanuelson U (2016). Herd-level factors associated with longevity in Swedish dairy cattle. Acta Agric Scand..

[22] Textor J, Hardt J, Knüppel S (2011). DAGitty: a graphical tool for analyzing causal diagrams. Epidemiology..

[23] Rubin DB (1987). Multiple imputation for nonresponse in surveys.

[24] Dohoo IR, Nielsen CR, Emanuelson U (2016). Multiple imputation in veterinary epidemiological studies: a case study and simulation. Prev Vet Med..

[25] Kudahl AB, Nielsen SS, Østergaard S (2011). Strategies for time of culling in control of paratuberculosis in dairy herds. J Dairy Sci.

[26] Nicholas RAJ, Fox LK, Lysnyansky I (2016). Mycoplasma mastitis in cattle: To cull or not to cull. Vet J..

[27] Svensson C, Alvåsen K, Eldh AC, Frössling J, Lomander H (2018). Veterinary herd health management—experience among farmers and farm managers in Swedish dairy production. Prev Vet Med..

[28] Kaler J, Green LE (2013). Sheep farmer opinions on the current and future role of veterinarians in flock health management on sheep farms: a qualitative study. Prev Vet Med..

[29] Duval JE, Bareille N, Fourichon C, Madouasse A, Vaarst M (2016). Perceptions of French private veterinary practitioners’ on their role in organic dairy farms and opportunities to improve their advisory services for organic dairy farmers. Prev Vet Med..

[30] Ruston A, Shortall O, Green M, Brennan M, Wapenaar W, Kaler J (2016). Challenges facing the farm animal veterinary profession in England: a qualitative study of veterinarians’ perceptions and responses. Prev Vet Med..

[31] Bergeå H, Roth A, Emanuelson U, Agenäs S (2016). Farmer awareness of cow longevity and implications for decision-making at farm level. Acta Agric Scand..

[32] De Vries A (2017). Economic trade-offs between genetic improvement and longevity in dairy cattle. J Dairy Sci.

[33] Fetrow J, Nordlund KV, Norman HD (2006). Invited review: culling: nomenclature, definitions, and recommendations. J Dairy Sci.

[34] Beaudeau F, Seegers H, Ducrocq V, Fourichon C, Bareille N (2000). Effect of health disorders on culling in dairy cows: a review and a critical discussion. Ann Zootech..

[35] Ahlman T, Ljung M, Rydhmer L, Röcklinsberg H, Strandberg E, Wallenbeck A (2014). Differences in preferences for breeding traits between organic and conventional dairy producers in Sweden. Livest Sci..

