# Supplementary material for: Farm characteristics and management routines related to cow longevity: a survey among Swedish dairy farmers
Source: Acta Vet Scand. 2018 Jun 19;60:38. doi: 10.1186/s13028-018-0390-8 (PMC6006783; doi:10.1186/s13028-018-0390-8)
Supplement: Supplementary file 1 — Additional file 1. An English version of the questionnaire that was sent to Swedish dairy farmers. [file 13028_2018_390_MOESM1_ESM.docx]

**Additional file 1.** An English version of the questionnaire that was sent to Swedish dairy farmers

#### Questionnaire about longevity in dairy cows

**QUESTIONS ABOUT THE FARM**

**Number of milking dairy cows: ___________**

1. **About me:**

🞏 Female

🞏 Male

Age: ______years

Role on the farm: ________________________

Are you the person in charge of the milking cows?

🞏 Yes 🞏 No

Proportion of the household’s [disposable income](http://tyda.se/search/disposable%20hypodermic%20syringe?w_lang=en) from milk production: __________%

Work experience in milk production: ______years

What level of education do you have?

🞏 Secondary school or equivalent

🞏 Agricultural college

🞏 Other college

🞏 Further education courses (within agriculture)

🞏 Agricultural technologist education

🞏 Agronomist education

🞏 Other university education

🞏 Other What? ­____________________

1. **How much do you appreciate doing the following work?** *Cross on the line*

Machinery maintenance

less fun fun

Crop production

less fun fun

Animal care

less fun fun

Feeding of animals

less fun fun

Milking

less fun fun

1. **Are you considering changing the number of cows in your herd over the next two or three years?**

🞏 Yes, I want to have 🞏 more cows 🞏 fewer cows

🞏 No

Comment:

1. **Is there anyone else taking care of the cows in your herd?**

🞏 No, **pass on to question 6**

🞏 Yes, only family member **pass on to question 6**

🞏 Yes, only employees

🞏 Yes, family member and employees

1. **How many employees (excluding family members)** **are caring for animals in the herd?**

🞏 1

🞏 2

🞏 3 or more

1. **Which milking system is used?**

🞏 Tie-stall pipeline

🞏 Parlor

🞏 Automatic milking system Brand:_________________________

🞏 Rotary

Comment:

1. **In what housing system are the majority of the milking cows kept?**

🞏 Tie stall

🞏 Free stall with cubicles

🞏 Free stall with deep straw bedding

🞏 Combination of tie stall and free stall

1. **Have you used any advisory services during the last 12 months?**

Feed 🞏 1-6 times 🞏 7 times or more 🞏 No

Preventive animal herd health 🞏 1-6 times 🞏 7 times or more 🞏 No

Breeding 🞏 1-6 times 🞏 7 times or more 🞏 No

1. **To your knowledge, will the farm still be running in five years?** *Cross on the line*

No Maybe Yes, for sure

🞏 Do not know

Comment:

**QUESTIONS ABOUT HOUSING**

1. **What type of floor is in the majority of the cubicles or cow places?**

Concrete: ______ % of the cubicles

Rubber: ______ % of the cubicles

Mattress: ______ % of the cubicles

Deep straw bedding: ______ % of the cow places

Comment:

1. **How many cubicles/cow places are there in the shed for the milking cows?**

______ (alternatively ______m^2^ of deep straw bedding)

1. **How is the feeding limited for the milking cows in the herd?**

🞏 Feed yokes (lockable or not) number of feed yokes: _________

🞏 No limitation (e.g. head rail) _________ meters of feed trough

Comment:

**If you have a tie stall: move on to question 16**

1. **How wide are the walkways behind an eating cow at the feed trough (yellow circle)?**


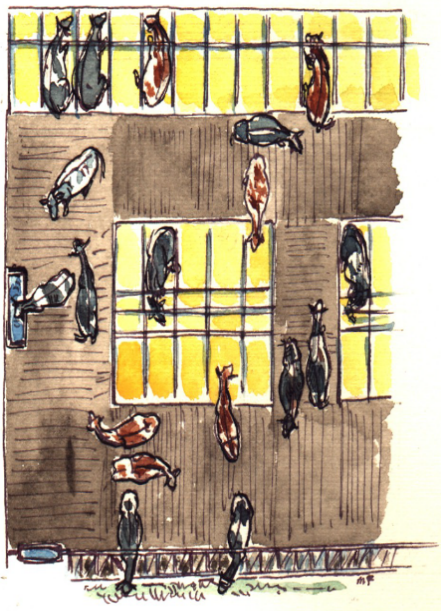


🞏 No cow can pass

🞏 1 cow can pass (blue circle)

🞏 2 cows can pass abreast (red circle)

🞏 More than 2 cows can pass abreast

Total width of the walkways behind the feed trough _______ meters (black arrow)

1. **What type of flooring is the majority of the walkways for the milking cows?**

🞏 Concrete

🞏 Slats

🞏 Rubber rugs - proportion of the shed: ≤ 25% 🞏 25-75% 🞏 ≥75% 🞏

Comment:

1. **How do you group your cows?** *Indicate all that apply*

🞏 Lactation stage

🞏 New-calved cows separately Number of days in this group _______

🞏 First parity cows separately

🞏 Pregnant/non pregnant

🞏 Somatic cell count/udder health

🞏 Lame cows

🞏 Fattening/slaughter cows

🞏 Treated cows with waiting period

🞏 Dry cows

🞏 Other grouping: ________________________

Comment:

**QUESTIONS ABOUT FEED**

1. **How many hours per day do the cows have access to feed (roughage or mixed feed) on the feed trough?**

__________hours/day

Comment:

1. **How many times per day are the cows fed with roughage or mixed feed?**

**___________**times/day

Comment:

1. **Do you check the troughs for remaining feed that is out of reach of the cows?**

🞏 Yes 🞏 1 time/day 🞏 2 times/day 🞏 >2 times/day

🞏 No

🞏 Irrelevant, the cows can always reach the feed

Comment:

1. **How large a grazing area is used for the milking cows?**

🞏 Production pasture __________ hectares

🞏 Exercise pasture __________ hectares

Comment:

1. **How many hours per day do the milking cows have access to pasture (during the most intensive pasture period)?**

____________hours/day

Comment:

1. **When during the day are the milking cows out on pasture?**

🞏 Only daytime

🞏 Only nighttime

🞏 Daytime and nighttime

🞏 It varies

Comment:

1. **Do the cows have the opportunity to go back into the shed whenever they want when out on pasture?**

🞏 Yes

🞏 Yes, sometimes When?______________________

🞏 No, the gates are shut

Comment:

1. **Are special feed rations calculated for cows in the transition period (the month before calving to the time soon after calving)?**

🞏 Yes, at least once a year

🞏 Yes, but not every year

🞏 No

1. **Are feed rations calculated for the heifers?**

🞏 Yes, at least once a year

🞏 Yes, but not every year

🞏 No

🞏 Do not raise heifers

1. **Do you analyze your roughage?**

🞏 Yes, for every harvest

🞏 Yes, at least once a year

🞏 Yes, but less than every year

🞏 No

Comment:

**QUESTIONS ABOUT ROUTINES**

1. **Are written herd health plans and daily routines used at the farm?**

🞏 Yes In which areas? ____________________________________________

🞏 No

Comment:

1. **How many minutes per day are used for surveillance?** Estimate the total amount of time for all persons among the milking cows (as well as heifers and calves) including milking, feeding, heat rounds, finding unhealthy/suspect animals, improving bedding, removing manure, etc.

Cows ___________min/day

Heifers ___________min/day

Calves ___________min/day

🞏 We use an activity meter system for 🞏 heifers 🞏 milking cows

🞏 We use alarm lists

Comment:

1. **Do you assess body condition score for heifers (6 months to 1st calving) and cows?**

Heifers: 🞏 Yes, regularly 🞏 Yes, sometimes 🞏 No

Cows: 🞏 Yes, regularly 🞏 Yes, sometimes 🞏 No

Comment:

1. **How many times each year is a cow’s hoof trimmed on average?**

🞏 1 🞏 2 🞏 3 or more

Comment:

1. **Is the whole herd trimmed at the same occasion?**

🞏 Yes (≥ 90%) 🞏 and some cows when needed

🞏 No What proportion are trimmed at the same occasion? 🞏 ≤50% 🞏 50-90%

Comment:

1. **Do you have access to a chute on the farm?**

🞏 Yes 🞏 No

Comment:

1. **How do the calves get their first colostrum?**

🞏 Manually Proportion of calves:___________%

🞏 Suckles themselves Proportion of calves:___________%

**Sum: 100 %**

Comment:

1. **Within how many hours after calving do the calves get colostrum, and how much colostrum do they get on average?**

**________** hours after calving

________ liters

1. **How many times do the young stock change system (e.g. individual pen, group pen on deep straw bedding, group pen on slats, free stall with cubicles, tie stall) until first calving?**

🞏 2 times

🞏 3 times

🞏 4 times

🞏 5 times or more

Comment:

1. **Where do the calvings occur?**

🞏 Group maternity pen with place for ___________cows/pen _______ pens

🞏 Individual maternity pen _______ pens

🞏 Not separated from the other cows

🞏 Outside

1. **For how long are the cow and calf together in the maternity pen (hours or days after calving)?**

___________hours *or*

___________days

🞏 Not relevant

Comment:

1. **Which type of bedding has been used for the milking cows during the last year?**

*Indicate all that apply.*

🞏 Sawdust

🞏 Cutter shavings

🞏 Peat

🞏 Whole straw

🞏 Chopped straw

🞏 Mix of _____________________________________________

🞏 Have changed bedding during the period

Comment:

1. **How often do you improve the bedding for the milking cows?**

_______times per 🞏 day 🞏 week 🞏 month

Comment:

1. **Are pregnant heifers kept with milking cows?**

🞏 Yes For how long? _________days before calving

🞏 No

Comment:

1. **When do you start to dry-off a cow?**

___________ days before scheduled calving

Comment:

1. **How long does it usually take to dry-off a cow in your herd?**

___________days

Comment:

1. **Are cows with high yield (at time of drying off) dried off later than the other cows?**

🞏 Yes 🞏 No

Comment:

1. **What do you think of breeding (i.e. genetic selection)?** *Cross on the line*

not so important very important

1. **Do you use a bull (natural service) in your herd?**

🞏 Yes To which groups of animals? Heifers 🞏 Cows 🞏

🞏 No

Comment:

1. **Which one of the following interventions is most important for reducing the proportion of cows that die unassisted or are euthanized in the herd?** *Choose one alternative*

🞏 Building technology

🞏 Higher carcass prices

🞏 Better disease control/biosecurity

🞏 Better genetic material

🞏 No special intervention, it mostly depends on good or bad luck

🞏 Not relevant

Comment:

**QUESTIONS ABOUT LAME AND SICK COWS**

1. **How do you recognize that a cow is lame?** *Cross on the lines*

**Does not put pressure on the leg when standing**

not so important very important

**Does not put pressure on the leg when walking**

not so important very important

**Hunching when standing**

not so important very important

**Hunching when walking**

not so important very important

**Increased lying time**

not so important very important

**Difficulties when standing up or lying down**

not so important very important

**Does not come to milking**

not so important very important

Comment:

1. **What is the first thing you do when you recognize a lame cow?** *Cross on the lines*

**Wait**

not so likely very likely

**Call the veterinarian**

not so likely very likely

**Call the hoof trimmer**

not so likely very likely

**Put the cow on a surveillance list**

not so likely very likely

**Move the cow to an isolation pen**

not so likely very likely

**Take her to the chute for hoof trimming**

not so likely very likely

**Start a treatment on my own**

not so likely very likely

1. **How do you recognize an unhealthy cow?** *Cross on the lines*

**Lying a lot**

not so important very important

**Changes in the milk**

not so important very important

**Swollen udder**

not so important very important

**Diarrhea**

not so important very important

**Nasal discharge**

not so important very important

**Stopped eating**

not so important very important

**Vaginal discharge**

not so important very important

**Fever, warm**

not so important very important

**Does not come for milking**

not so important very important

**Reduced general condition**

not so important very important

Comment:

1. **What is the first thing you do when you recognize an unhealthy cow (excluding accidents, e.g. fracture of a leg)?** *Cross on the lines*

**Wait**

not so likely very likely

**Measure her temperature**

not so likely very likely

**Call the veterinarian**

not so likely very likely

**Add her to a surveillance list**

not so likely very likely

**Move the cow to an isolation pen**

not so likely very likely

**Start a treatment on my own**

not so likely very likely
