# Supplementary material for: Farm characteristics and management routines related to cow longevity: a survey among Swedish dairy farmers
Source: Acta Vet Scand. 2018 Jun 19;60:38. doi: 10.1186/s13028-018-0390-8 (PMC6006783; doi:10.1186/s13028-018-0390-8)
Supplement: Supplementary file 2 — Additional file 2. Continuous variables used in the analysis of factors related to average herd longevity in 228 dairy herds in Sweden. Descriptive statistics and P-values of associations with herd average longevity are presented. [file 13028_2018_390_MOESM2_ESM.docx]

**Additional file 2.** Continuous variables used in the analysis of factors related to average herd longevity in 228 dairy herds in Sweden. Descriptive statistics and P-values of associations^1^ with herd average longevity are presented.

| Variable | n^2^ | Continuous variables | P-value | Question number^3^ |
| --- | --- | --- | --- | --- |
|  |  | Mean, SD (min, max) |  |  |
| Average herd longevity (d) (outcome of interest) | 228 | 1581.7, 293.3  (475, 2555) | n.a. | n.a. |
| Herd size (number of milking cows) | 213 | 87.6, 84.6  (26, 1000) | 0.192 | Q0 |
| Age of farmer (yr) | 225 | 48.7, 11.3  (20, 79) | <0.001 | Q1 |
| % income from milk production | 202 | 72.0, 26.1  (0, 100) | 0.928 | Q1 |
| Years in milk production | 221 | 27.7, 12.9  (2, 60) | 0.764 | Q1 |
| Interest in machines | 225 | 4.5, 2.7  (0.1, 9.8) | 0.381 | Q2 |
| Interest in crop production | 225 | 6.1, 2.5  (0, 10) | 0.607 | Q2 |
| Interest in animal care | 228 | 8.0, 1.7  (1.2, 10) | 0.787 | Q2 |
| Interest in feeding of animals | 228 | 7.5, 1.8  (0.5, 10) | 0.169 | Q2 |
| Interest in milking | 228 | 7.3, 2.2  (0.2, 10) | 0.950 | Q2 |
| Probability that the farm will still be operating in 5 yr | 227 | 7.6, 2.7  (0, 10) | 0.144 | Q9 |
| Access to feed (h) | 208 | 20.8, 4.8  (5, 24) | 0.263 | Q16 |
| Number of feedings per d | 211 | 4.9, 3.8  (0.5, 24) | 0.516 | Q17 |
| Access to pasture (h/d) | 211 | 16.2, 6.4  (6, 24) | 0.946 | Q20 |
| When drying off is initiated (d before calving) | 217 | 63.9, 8.2  (40, 90) | 0.461 | Q40 |
| Average time for dry-off (d) | 214 | 7.8, 4.3  (0, 21) | 0.119 | Q41 |
| Interest in animal breeding (i.e. genetic selection) | 223 | 7.9, 2.0  (0.5, 10) | 0.145 | Q43 |
| Recognize a lame cow  (Cronbach’s α) | 220 | 8.1, 1.3  (4.32, 10) | 0.988 | Q46 |
| Wait when recognizing  a lame cow | 215 | 3.6, 2.8  (0, 10) | 0.430 | Q47 |
| Call veterinarian when recognizing a lame cow | 210 | 4.4, 3.1  (0, 10) | 0.003 | Q47 |
| Call hoof trimmer when recognizing a lame cow | 206 | 4.6, 3.2  (0, 10) | 0.456 | Q47 |
| Put on surveillance list when recognizing a lame cow | 203 | 5.6, 3.7  (0, 10) | 0.581 | Q47 |
| Move to isolation pen when recognizing a lame cow | 202 | 5.3, 3.2  (0, 10) | 0.029 | Q47 |
| Take to chute when recognizing a lame cow | 202 | 6.0, 3.5  (0, 10) | 0.215 | Q47 |
| Start treatment when recognizing a lame cow | 202 | 5.5, 3.4  (0, 10) | 0.636 | Q47 |
| Detection of unhealthy cows (Cronbach’s α) | 216 | 8.0, 1.2  (4.2, 10) | 0.437 | Q48 |
| Wait when recognizing unhealthy cows | 213 | 2.7, 2.6  (0, 10) | 0.706 | Q49 |
| Measure temp when recognizing unhealthy cows | 216 | 8.3, 2.1  (0.2, 10) | 0.472 | Q49 |
| Call veterinarian when recognizing unhealthy cows | 219 | 7.5, 2.4  (0.1, 10) | 0.011 | Q49 |
| Put on surveillance list when recognizing unhealthy cows | 209 | 5.9, 3.7  (0, 10) | 0.877 | Q49 |
| Move to isolation pen when recognizing unhealthy cows | 213 | 6.1, 3.2  (0, 10) | 0.121 | Q49 |
| Start treatment when recognizing unhealthy cows | 215 | 5.0, 3.4  (0, 10) | 0.148 | Q49 |

^1^ For all associations except for region, number of cows, and gender, which were forced into the model as potential confounders

^2^ Number of observations before multiple imputation was done

^3^ Question number refers to the questionnaire that can be found in Additional file 1
