# Supplementary material for: Farm characteristics and management routines related to cow longevity: a survey among Swedish dairy farmers
Source: Acta Vet Scand. 2018 Jun 19;60:38. doi: 10.1186/s13028-018-0390-8 (PMC6006783; doi:10.1186/s13028-018-0390-8)
Supplement: Supplementary file 3 — Additional file 3. Categorical variables used in the analysis of factors related to average herd longevity in 228 dairy herds in Sweden. Descriptive statistics and P-values of associations with average herd longevity are presented. [file 13028_2018_390_MOESM3_ESM.docx]

**Additional file 3.** Categorical variables used in the analysis of factors related to average herd longevity in 228 dairy herds in Sweden. Descriptive statistics and P-values of associations^1^ with average herd longevity are presented.

| Variable | n^2^ | Categorical variables | | P-value | Question number^3^ |
| --- | --- | --- | --- | --- | --- |
|  |  | Categories | n (%) |  |  |
| Geographical region of Sweden | 228 | South Sweden | 32 (14) | 0.089 |  |
|  |  | East Sweden | 62 (27) |  |  |
|  |  | West Sweden | 74 (32) |  |  |
|  |  | Middle Sweden | 20 (9) |  |  |
|  |  | North Sweden | 40 (18) |  |  |
| Gender | 223 | Female | 64 (29) | 0.520 | Q1 |
|  |  | Male | 159 (71) |  |  |
| In charge of milking | 212 | No | 24 (11) | 0.337 | Q1 |
|  |  | Yes | 188 (89) |  |  |
| Education level | 224 | Secondary school | 35 (16) | 0.941 | Q1 |
|  |  | College | 94 (42) |  |  |
|  |  | Agricultural courses | 63 (28) |  |  |
|  |  | University | 32(14) |  |  |
| Changing the number of milking cows | 224 | Same or fewer | 163 (73) | 0.046 | Q3 |
|  |  | Expand | 61 (27) |  |  |
| Composition of workforce | 222 | Family | 94 (42) | 0.141 | Q4 |
|  |  | Employees | 128 (58) |  |  |
| Milking system | 224 | Tie-stall pipeline | 104 (46) | 0.382 | Q6 |
|  |  | parlor/rotary | 44 (20) |  |  |
|  |  | AMS | 76 (34) |  |  |
| Housing | 227 | Tie stall | 127 (56) | 0.070 | Q7 |
|  |  | Free stall | 100 (44) |  |  |
| Used feed advisory  services | 216 | Never | 48 (22) | 0.970 | Q8 |
|  |  | Sometimes | 168 (78) |  |  |
| Used preventive herd health advisory services | 182 | Never | 82 (45) | 0.029 | Q8 |
|  |  | Sometimes | 100 (55) |  |  |
| Use of breeding advisory services | 204 | No | 55 (27) | 0.123 | Q8 |
|  |  | Yes | 149 (73) |  |  |
| Type of pasture | 209 | Production | 69 (33) | 0.816 | Q19 |
|  |  | Exercise | 140 (67) |  |  |
| Only access to pasture during day-time | 217 | Day only | 57 (26) | 0.996 | Q21 |
|  |  | Other | 160 (74) |  |  |
| Closed out on pasture | 219 | No | 114 (52) | 0.230 | Q22 |
|  |  | Yes, they can return to the shed | 105 (48) |  |  |
| Special feed rations for cows in transition period | 212 | No | 97 (46) | 0.187 | Q23 |
|  |  | Yes | 115 (54) |  |  |
| Calculated feed rations for heifers | 213 | No | 97 (46) | 0.155 | Q24 |
|  |  | Yes | 115 (54) |  |  |
| Performing analysis of the roughage | 219 | <1 time/yr | 29 (13) | 0.556 | Q25 |
|  |  | ≥1 time/yr | 190 (87) |  |  |
| Use of written herd health plans and daily routines | 218 | Yes | 148 (68) | 0.831 | Q26 |
|  |  | No | 70 (32) |  |  |
| Assessment of body condition score for heifers | 213 | No | 67 (31) | 0.251 | Q28 |
|  |  | Yes, sometimes | 87 (41) |  |  |
|  |  | Yes, regularly | 59 (28) |  |  |
| Assessment of body condition score for cows | 216 | No | 60 (28) | 0.632 | Q28 |
|  |  | Yes, sometimes | 86 (40) |  |  |
|  |  | Yes, regularly | 70 (32) |  |  |
| Number of hoof trims per yr | 214 | Once | 31 (14) | 0.707 | Q29 |
|  |  | 2 times | 158 (74) |  |  |
|  |  | 3 or more | 25 (12) |  |  |
| Is the whole herd trimmed at the same occasion? | 217 | Yes | 101 (47) | 0.298 | Q30 |
|  |  | Yes, and some when needed | 89 (41) |  |  |
|  |  | No, a small proportion of the cows | 27 (12) |  |  |
| Access to a crush on the farm | 219 | Yes | 111 (51) | 0.686 | Q31 |
|  |  | No | 108 (49) |  |  |
| Number of times the young stock change systems | 197 | 2 | 38 (19) | 0.972 | Q34 |
|  |  | 3 | 84 (43) |  |  |
|  |  | 4 | 46 (23) |  |  |
|  |  | ≥5 | 29 (15) |  |  |
| Calvings occur in individual maternity pens | 227 | Yes, only | 78 (34) | 0.017 | Q35 |
|  |  | No, other | 149 (66) |  |  |
| Time cow and calf are kept together before separated | 171 | <24h | 114 (67) | 0.220 | Q36 |
|  |  | >24h | 57 (33) |  |  |
| Type of bedding | 220 | Wood/sawdust | 123 (56) | 0.737 | Q37 |
|  |  | Straw | 44 (20) |  |  |
|  |  | Other | 53 (24) |  |  |
| Daily improvement of the bedding? | 199 | Yes | 150 (75) | 0.347 | Q38 |
|  |  | No, less frequent | 49 (25) |  |  |
| Pregnant heifers are kept with the milking cows | 217 | Yes | 141 (56) | 0.505 | Q39 |
|  |  | No | 76 (35) |  |  |
| High yielders (at time for drying off) are dried off later than other cows | 219 | Yes | 61 (28) | 0.504 | Q42 |
|  |  | No | 158 (72) |  |  |
| Natural service (bull) used | 223 | Yes  No | 96 (43)  127 (57) | 0.729 | Q44 |

^1^For all associations except for region, number of cows, and gender, which were forced into the model as potential confounders

^2^ Number of observations before multiple imputation was done

^3^ Question number refers to the questionnaire that can be found in Additional file 1
